# Gender Differences in Treatment Outcomes for Eating Disorders: A Case-Matched, Retrospective Pre–Post Comparison

**DOI:** 10.3390/nu14112240

**Published:** 2022-05-27

**Authors:** Georg Halbeisen, Karsten Braks, Thomas J. Huber, Georgios Paslakis

**Affiliations:** 1 University Clinic for Psychosomatic Medicine and Psychotherapy, Medical Faculty, Campus East-Westphalia, Ruhr-University Bochum, 32312 Luebbecke, Germany; georg.halbeisen@rub.de; 2 Centre for Eating Disorders, Klinik am Korso, 32545 Bad Oeynhausen, Germany; karsten.braks@korso.johanniter-kliniken.de (K.B.); thomas.huber@korso.johanniter-kliniken.de (T.J.H.)

**Keywords:** anorexia nervosa, bulimia nervosa, binge eating disorder, eating disorder, men’s health, diversity, psychotherapy

## Abstract

Eating disorders (EDs) are increasingly emerging as a health risk in men, yet men remain underrepresented in ED research, including interventional trials. This underrepresentation of men may have facilitated the development of women-centered ED treatments that result in suboptimal outcomes for men. The present study retrospectively compared pre- vs. post-treatment outcomes between age-, diagnosis-, and length-of-treatment-matched samples of *n* = 200 men and *n* = 200 women with Anorexia Nervosa (AN), Bulimia Nervosa (BN), Binge Eating Disorder (BED), or Eating Disorder Not Otherwise Specified (EDNOS), treated in the same setting during the same period, and using the same measurements. Compared to women, men with AN showed marked improvements in weight gains during treatment as well as in ED-specific cognitions and general psychopathology. Likewise, men with BED showed marked weight loss during treatment compared to women with BED; ED-specific cognitions and general psychopathology outcomes were comparable in this case. For BN and EDNOS, weight, ED-specific cognitions, and general psychopathology outcomes remained largely comparable between men and women. Implications for treatments are discussed.

## 1. Introduction

Eating disorders (EDs) are of increasing public health concern [1]. Characterized by body image disturbances, abnormal eating patterns, and weight-control behaviors [2], an estimated 2.6 to 8.4% (women) and 0.7 to 2.2% (men) of the global population suffer from Anorexia Nervosa (AN), Bulimia Nervosa (BN), Binge Eating Disorder (BED), and other EDs during their lifetime [3,4,5]. EDs pose one of the highest mortality risks among mental disorders [6] and are associated with adverse physical and mental health outcomes across multiple domains of functioning [7]. Globally, the disability-adjusted life years (DALYs) for EDs amount to 43.36 per 100,000 individuals, with data for Western Europe suggesting a burden of 112.27 DALYs [8]. The healthcare and economic costs of untreated EDs are substantial [9], emphasizing the importance of tailoring treatments toward the needs of patients with EDs.

Counter to the widespread perception that EDs primarily affect adolescent girls and women [10,11], EDs increasingly emerge as a health risk in men [12,13,14]. Although overall still lower, men’s prevalence rates have increased faster than women’s prevalence rates since 1990 (by 22% vs. 12% to 117.9 vs. 231.5 per 100,000 men and women in 2019, respectively [15]). Similarly, men’s DALYs increased by 0.70% annually, compared to a 0.63% annual increase for women [8]. These findings suggest that men could make up every third clinical ED case, although there is agreement that the available data still underestimate men’s prevalence and burden due to men hesitating to disclose their ED and access treatment [16,17].

Men also remain underrepresented in ED research [18], suggesting potential gender biases in the development of ED-specific diagnostic criteria and assessment tools [19]. The changing demographics of the ED population thus sparked a considerable effort to further the understanding of gender differences in ED presentation and etiology [20,21]. Studies reveal that men with EDs, compared to women, are often less concerned with thinness [22,23,24], seek to increase body mass and muscularity instead [25,26], and may also present with different patterns in emotion regulation [27]. Given that established assessment tools rarely address men-associated ED symptoms, recent developments include men-oriented norms and extensions for established ED questionnaires [28,29,30] as well as men-specific assessment instruments [31,32,33,34].

Speculations persist that men’s underrepresentation may have also facilitated the development of women-centered ED treatments [26], resulting in suboptimal outcomes for men. Evidence-based therapeutic recommendations for EDs include nutritional rehabilitation and psychotherapy [35] and primarily derive from interventional trials that average between 7.8 and 16.3% men [18,36]. For example, among the more than 300 trials included in the German ED treatment guidelines [37], men comprise only 10%, 5%, and less than 1% of treatment samples for BED, AN, and BN, respectively [13]. A systematic review of qualitative studies on men’s treatment experiences corroborates the potential adverse effects of this level of underrepresentation [14]. Due to the perceived femininity of EDs [38], men may delay help-seeking and present later in the course of their illness when pathology is more severe and less tractable to intervention [39]. When men seek treatment, they may struggle to feel understood by therapists [40], feel unwanted in the treatment environment [41], experience marginalization in otherwise women-dominated facilities [42], or feel that men-specific concerns are not adequately addressed [41].

Thus far, however, the evidence on gender differences or parity in ED treatment outcomes remains limited and inconclusive [43,44]. Among adolescent patients with EDs admitted to inpatient treatment, Coelho et al. [45] found more favorable outcomes for 20 adolescent males (14 AN, 6 other) compared to 20 females (12 AN, 1 BN, 7 other) in terms of ED-related cognitions. Nagata et al. [46] observed greater weight change among 95 hospitalized adolescent males (58 AN, 1 BN, 36 other) compared to 493 females (363 AN, 8 BN, 1 BED, 121 other), but also that male adolescents required a longer length of stay than females. However, Gorrell et al. [47] compared gender differences in treatment response using baseline and end-of-treatment data from two clinical trials with adolescent patients with AN (24 male, 204 female) and found no significant gender differences for either weight gains or changes in ED-related cognitions.

Study findings comparing treatment outcomes among hospitalized adult patients with EDs similarly remain inconclusive. In a matched comparison of 131 men and 131 women with EDs, Agüera et al. [48] observed a higher risk for dropout from treatment for men than women with BN but also higher remission rates among men compared to women with OSFED (i.e., for patients with Otherwise Specified Feeding and Eating Disorder according to Diagnostic and Statistical Manual of Mental Disorders, Fifth Edition (DSM-5) criteria). Findings from Fernández-Aranda et al. [49] instead suggest more favorable outcomes in terms of ED-related cognitions among 19 men compared to 150 women with BN following group psychotherapy. Lydecker et al. [50] further observed improved weight loss but similar outcomes in ED-related cognitions when comparing trial data from 170 men against 490 women with BED. Two studies comparing long-term outcomes between men and women with EDs provide further mixed findings, with one study showing more favorable outcomes among 157 men with AN but not among 83 men with BN, compared to matched samples of women with EDs [51], and another study showing shorter long-term survival within the same population of men with AN and BN compared to women [52].

Given the overall paucity of evidence, often insufficient sample sizes of men, and rare use of gender-matched controls, further research on gender differences in ED treatment is urgently needed [13,14,43]. Thus, the goal of the current study was to advance our understanding of gender differences in ED treatment outcomes by systematically comparing data from adult men and women with EDs admitted to inpatient treatment. Specifically, routine data covering admission, treatment, and end-of-treatment, consecutively collected over four years at a German clinic specialized in the treatment of EDs, were used to construct, evaluate, and compare weight trajectories, changes in ED-related cognitions, and general psychopathology between gender- and diagnosis-matched ED patient samples. We refrained from making strong predictions about gender differences in ED treatment outcomes due to the absence of conclusive evidence. Instead, we formulated a working hypothesis based on qualitative evidence [14] that when controlling for baseline and treatment characteristics, men with EDs would show weaker treatment responses than women with EDs. We evaluated the strength of evidence for and against the working hypothesis within different outcome domains.

## 2. Materials and Methods

### 2.1. Participants

A total of 47 men with AN, 18 men with BN, 125 men with BED, and 10 men with Eating Disorder Not Otherwise Specified (EDNOS) according to International Classification of Diseases, 10th Revision (ICD-10) criteria were admitted and consecutively treated at least once between January 2018 and December 2021 at the Klinik am Korso, Bad Oeynhausen, Germany. Inpatient treatment followed a multimodal rehabilitation concept based on depth psychology and cognitive–behavioral approaches and included individual and group psychotherapy, psychoeducation, nutritional rehabilitation, and complementary therapies (e.g., occupational therapy). Therapists were trained physicians and psychologists who participated in regular supervision.

All 200 men with EDs were included in this study. For men treated more than once in the clinic (2 AN, 1 BED), only the first admission data were chosen for analysis. Similar to Strobel et al. [51], three criteria, as explained below, matched each man to one first admission dataset from a large sample of women with EDs treated at the same clinic during the same time (908 women with AN, 622 women with BN, 388 women with BED, and 40 women with EDNOS). Men were matched to women of the same ED diagnosis (and diagnosis subtype) of AN, BN, BED, or EDNOS (Criterion 1), resulting in a perfect match of diagnoses. Within each diagnostic group, for each man, a woman with the nearest value of age at admission was selected (Criterion 2), followed by selecting the person with the closest length of treatment duration (Criterion 3).

The study was reviewed and approved by the Ethics Committee of the Ruhr-University Bochum’s Medical Faculty at Campus East-Westphalia as part of application AZ 2021-849, and prospectively registered with the German Clinical Trial Register as part of application DRKS00028441. Datasets are available from the corresponding author upon reasonable request.

### 2.2. Outcome Assessments

#### 2.2.1. Patient Characteristics and Body Weight

Age, gender, and body height (in cm) were assessed for all patients at admission. Bodyweight (in kg) was assessed at admission, daily during the first week of admission, and once per week starting with the second week of admission. In addition, all patients were asked to complete a battery of standardized assessments at admission and end-of-treatment, detailed below.

#### 2.2.2. Eating Disorder Symptoms

Cognitive and behavioral symptoms of EDs were assessed using the Eating Disorder Examination-Questionnaire (EDE-Q). The EDE-Q [53] is a self-report questionnaire modeled after the Eating Disorder Examination interview [54]. The validated German translation [55,56] includes 23 items that assess how often ED symptoms occurred within the past 28 days on four subscales: Restraint (e.g., “On how many of the last 28 days…have you consciously tried to limit the amount of food you eat to affect your figure or weight?”), Eating Concern (e.g., “…has thinking about food, eating, or calories made it difficult for you to focus on things that interest you?”), Weight Concern (e.g., “…did you have a strong desire to lose weight?”), and Shape Concern (e.g., “…did you feel fat?”). Items were rated on a 7-point scale (from 1, never, up to 7, every day). Mean scores are computed for each subscale and a global score is computed for the overall questionnaire. Six additional open-ended questions assess the frequency of compensatory behaviors and objective binge episodes.

Patients’ body perception and body image were assessed using the FBeK (Fragebogen zur Beurteilung des eigenen Körpers), which is a widely used questionnaire in Germany for assessing individuals’ subjective views of their own bodies [57]. The FBeK includes 52 statements evaluated in a yes or no format and assesses body perception and body image on four subscales related to Physical Attractiveness and Self-confidence (e.g., “I am satisfied with my weight and with my size”), Accentuation of Physical Appearance (e.g., “I often and gladly look at myself”), Insecurities and Concerns related to bodily processes (e.g., “My body has a mind of its own”), and Physical/Sexual Discomfort (e.g., “I do not like being touched”). The manual provides gender-based percentile ranks of subscale means that were used for analysis.

#### 2.2.3. General Psychopathology

General psychopathology was assessed using the symptom checklist SCL-27-plus, a short, multidimensional screening instrument that contains 28 items across five subscales for depressive (e.g., “loss of joy”), vegetative (e.g., “nausea”), agoraphobic (e.g., “fear of leaving the house alone”), and sociophobic symptoms (e.g., “feeling of being unwanted”) as well as a subscale for pain (e.g., “headache”), a global symptom severity index, a lifetime assessment for depressive symptoms, and screening questions for suicidality [58]. Symptoms are rated on a 5-point scale (from 0, never, to 4, very often), with additional dichotomous ratings for lifetime depression (occurrence of depressive symptoms for more than two weeks), and frequency estimates for suicidal ideations and suicide attempts. Among patients with Eds, the SCL-27-plus mean scores have demonstrated good reliabilities and sensitivity to change [59].

The Beck Depression Inventory (BDI-II) [60] was also included as a widely used self-report inventory to measure the severity of depression in adults. The BDI-II contains 21-items, each scored on a 4-point scale, with sum scores ranging between 0 and 63.

### 2.3. Statistical Analysis

Patient and treatment characteristics were compared between ED and gender groups using univariate analysis of variance (ANOVA).

Weight outcomes were transformed to body mass index (BMI) (kg/m^2^) scores using patient admission height data. Similar to Strobel et al. [51], raw BMI values were further transformed into age- and gender-standardized z-scores using the lambda-mu-sigma (LMS) method [61] and German general population reference data [62,63]. Z-scores indicate the deviation of patient BMI relative to the population mean, allow extremes to be quantified outside the percentile range, and are comparable independent of age and sex [64]. We compared admission zBMI using independent samples *t*-test. Changes from admission to end-of-treatment and gender-based comparisons of zBMI change were analyzed using univariate analyses of covariance (ANCOVAs), with initial admission levels and length of treatment as co-variates. Weight trajectories, i.e., the timing-dependent changes from admission at different timepoints during treatment (zBMI_timepoint_–zBMI_admission_), were further analyzed as a function of gender, ED group, and treatment timepoint using linear mixed-effects (LME) modeling in R package lme4 Version 1.1.28 (R Core Team, Vienna, Austria) [65]. LME models describe an outcome as the linear combination of fixed effects, i.e., the independent predictors, and random effects, such as patient variance. They are ideally suited to analyze continuous data from mixed designs in which each case provides a differently sized dataset [66]. Models were fitted using restricted maximum likelihood (REML) estimation and built empirically using likelihood ratio tests for model comparisons via R’s ANOVA command. For model comparisons involving differences in fixed effects, models were refitted using maximum likelihood (ML) estimation. For parameter estimates of the fixed effects, *p*-values are based on Type III ANOVA as implemented in the R package car version 3.0.12 (R Core Team, Vienna, Austria) [67]. Pairwise comparisons used R package emmeans version 1.7.2 [68].

Patient questionnaire responses were aggregated according to each questionnaire’s specifications. Admission differences were evaluated using independent samples *t*-test. In case of violated assumptions about homoscedasticity, *t*-tests with adjusted degrees of freedom (df) are reported. Changes from admission to end-of-treatment, and gender-based comparisons of change, were analyzed using univariate ANCOVAs with initial admission levels and length of treatment as co-variates.

For a subsample of 104 men who provided information on previous external inpatient or outpatient treatment, we conducted an additional set of analyses, as described above, using a matched subsample of 104 women, and using the number of previous treatments as an additional covariate. However, because these analyses yielded descriptively similar findings concerning gender differences in treatment outcomes compared to the full sample—with deviations in inferential statistics due to reduced power—these analyses are not reported in detail here.

Descriptive results are reported as means and standard deviations (SDs). The significance level for all analyses was set at *p* ≤ 0.05. Post hoc pairwise comparisons report Bonferroni-adjusted *p*-values for multiple comparisons. Effect sizes are reported as *η^2^* and Cohen’s *d.* Instead of classical power calculation, which evaluates the strength of evidence against an arbitrarily defined effect, evidence strength for gender differences in treatment outcomes was evaluated using inclusion Bayes factor in Bayesian ANCOVA [69]. The inclusion Bayes factor provides a continuous measure of support for either H1 (gender modulates an outcome) or H0 (gender does not modulate an outcome) by quantifying the change from prior inclusion odds (i.e., the probability that gender is included as a predictor in a specific statistical model before seeing the data) to posterior inclusion odds (i.e., the probability of including gender in the statistical model after seeing the data). By convention, factors greater than three are considered as evidence for H1 and, vice versa, a Bayes factor smaller than 1/3 indicates evidence in favor of the null [70]. In other words, if the data are three times more likely with gender as a predictor than without gender in the model (BF_incl_ ≥ 3), the data support H1. If, however, the data are three times more likely in the absence of gender than in its presence (BF_incl_ ≤ 1/3), the data support rejecting H1 and accepting H0. Though any BF_incl_ > 1 supports H1 and any BF_incl_ < 1 supports H0, BF_incl_ ranging from 1/3 to 3 are considered “anecdotal”, suggesting that further research is needed.

Statistical analyses and case–control matching were conducted using SPSS Statistics version 28 for Windows (SPSS Inc., Chicago, IL, USA) [71]. Mixed models and plots were calculated in R version 4.1.3 (R Core Team, Vienna, Austria) [72]. Bayes factors were computed using Bayes ANCOVA in JASP version 0.16.1 (JASP Team, Amsterdam, The Netherlands) [73].

## 3. Results

### 3.1. Patient and Treatment Characteristics

Table 1 summarizes patients’ age and length of treatment. Consistent with the intended matching, two separate 2 (gender: men vs. women) × 4 (ED group: AN vs. BN vs. BED vs. EDNOS) ANOVAs revealed that gender groups were comparable in terms of age and length of treatment: gender main effect and gender × ED group interactions were not significant, all *F*s < 0.9, all *p*s > 0.36. All pairwise comparisons between gender groups for age and length of treatment were not significant, all *p*s > 0.09. There was only a main effect of ED group on age, *F*(3, 392) = 18.58, *p* < 0.001, *η^2^* = 0.12, with post hoc comparisons showing that patients with AN were overall younger compared to both patients with BN and BED, *p*s = 0.015 and <0.001; age means among all other groups remained comparable, all *p*s > 0.11.

### 3.2. Body Weight Outcomes

Appendix A Table A1 includes body weight outcomes (for additional statistics, see Appendix A Table A2). At admission, age- and gender-standardized BMI z-scores were comparable between men and women with AN, men and women with BN, and men and women with EDNOS, all |*t*|s < 1.50, all *p*s > 0.14. However, admission BMI z-scores were significantly higher in men than women with BED, *t*(238) = 9.69, *p* < 0.001, *d* = 1.23, 95% confidence interval (CI) (0.95, 1.50), suggesting more severe levels of age- and gender-adjusted obesity at the start of men’s treatment. At the end-of-treatment, BMI z-scores increased significantly for men and women with AN, *F*(1, 44) = 23.04, *p* < 0.001, *η^2^* = 0.34 and *F*(1, 44) = 9.18, *p* < 0.001, *η^2^* = 0.17, decreased for men and women with BED, *F*(1, 122) = 8.18, *p* < 0.001, *η^2^* = 0.06 and *F*(1, 122) = 6.72, *p* < 0.001, *η^2^* = 0.05, and remained comparable between men and women with BN and EDNOS, all *F*s < 4.49, *p*s > 0.07. Overall changes in BMI z-scores from admission to end-of-treatment, controlled for admission (baseline) levels and treatment duration differences, did not differ between gender groups for patients with AN, BN, and EDNOS, all *F*s < 3.3, *p*s > 0.08. However, men with BED showed more pronounced weight loss compared to women with BED, *F*(1, 246) = 96.50, *p* < 0.001, *η^2^* = 0.28.

**Figure 1:**
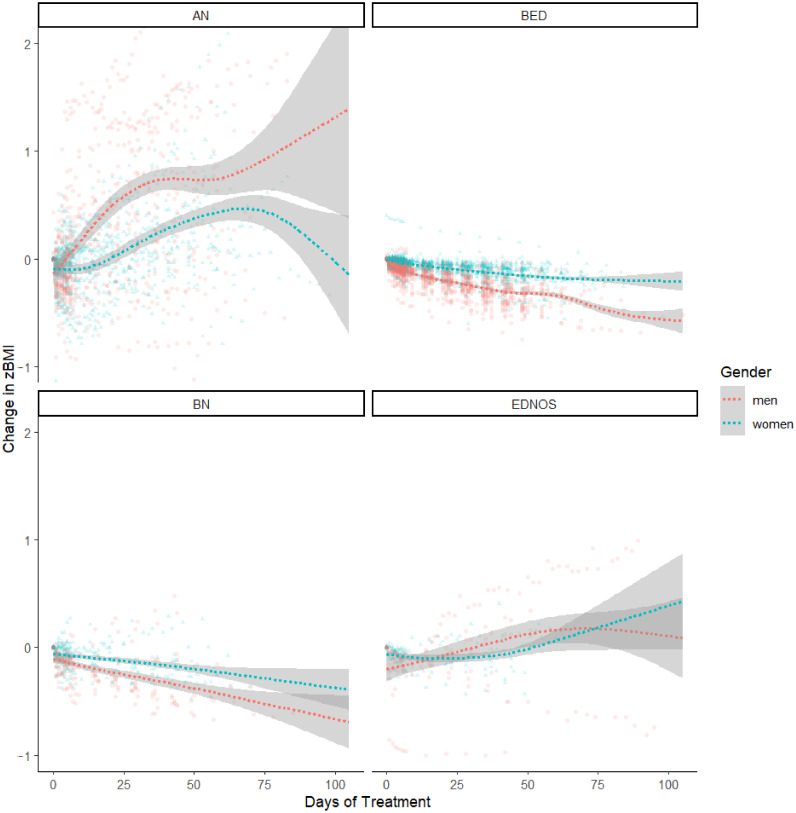
Patients’ weight trajectories, i.e., the time-dependent changes in age- and gender- standardized (zBMI) from admission to different timepoints during treatment (zBMI_timepoint_–zBMI_admission_), as a function of gender, ED group, and treatment timepoint. Curved regression lines (with 95% confidence bands) were fitted using function geom_smooth(), method (“gam”), as implemented in R package ggplot2 v. 3.3.5 (R Core Team, Vienna, Austria) [74]. ED = Eating Disorder, AN = Anorexia Nervosa, BN = Bulimia Nervosa, BED = Binge Eating Disorder, EDNOS = Eating Disorder Not Otherwise Specified, BMI = body mass index.

Figure 1 visualizes weight trajectories, i.e., the time-dependent changes from admission to different timepoints during treatment (zBMI_timepoint_–zBMI_admission_). LME modeling of changes in BMI z-scores as a function gender, ED group, and treatment timepoint started with constructing a null model using only participant identity as a random effect. Then, we sequentially added treatment timepoint (in days), ED group, and finally patient gender as fixed effects, allowing for all interactions. Each inclusion significantly improved the model’s goodness of fit, χ^2^(df = 1) = 8.83, *p* = 0.003, χ^2^(df = 6) = 2004.71, *p* < 0.001, and χ^2^(df = 8) = 144.44, *p* < 0.001, respectively.

The resulting model (see Table 2) revealed significant main effects for treatment timepoint, *p* < 0.001, ED group, *p* = 0.004, ED group × timepoint and gender × timepoint interactions, *p*s < 0.001, which were qualified by a significant timepoint × gender × ED group interaction, *p* < 0.001. We investigated the interaction further by comparing gender groups within each diagnostic category at every seventh day of treatment, starting at admission (timepoint = 0) and ending after nine weeks (timepoint = 63), at which 90% of patients had concluded their treatment. With increased temporal resolution compared to an ANCOVA (see above), the LME-based comparisons (see Appendix A Table A3) revealed a persistent and significant advantage in weight gain for men over women with AN after the first week of treatment and a persistent and significant advantage in weight loss in men over women with BED after the first week of treatment. Weight change in patients with BN remained comparable until seven weeks of treatment, at which point the model estimated more weight loss in men compared to women, although men remained within the overweight BMI range and women remained within the normal BMI range. No significant gender differences in weight change were estimated at any point during treatment for patients with EDNOS.

We further evaluated the significance of week-by-week changes in zBMI scores within each gender and ED group, using area under the curve formulae for time-dependent changes [75], to determine the timepoints at which weight changes occurred during treatment. LME-based estimates revealed significant week-by-week weight increases in men with AN over nine weeks of treatment, all *p*s < 0.001. Women with AN showed weight increases starting in week 4, until week 9 of treatment, all *p*s < 0.01. Men with BED showed weekly weight reductions between week 1 and week 7 of treatment, all *p*s < 0.03, whereas women with BED showed weekly weight reductions between week 1 and 5 of treatment, all *p*s < 0.003. For men with BN, weight reductions were observed between weeks 1 and 4, all *p*s < 0.01, whereas women with BN showed weight reduction between weeks 1 and 3, all *p*s < 0.05. Men with EDNOS showed weight reductions during weeks 1 and 2, *p*s < 0.05, and a weight increase in week 9, *p* = 0.03. Week-by-week changes were not detectable among women with EDNOS, all *p*s > 0.13.

### 3.3. Eating Disorder Symptoms’ Outcomes

Appendix A Table A1 includes the summary of EDE-Q outcomes. At admission, EDE-Q total scores and subscale scores for Restraint, Eating Concerns, Weight Concerns, and Shape Concerns were comparable between men and women with AN, all |*t*|s < 0.88, all *p*s > 0.39, men and women with BN, all |*t*|s < 0.57, all *p*s > 0.58, and men and women with EDNOS, all |*t*|s < 1.29, all *p*s > 0.22. However, men with BED had lower EDE-Q total and subscale scores than women with BED, all *t*s < −2.42, all *p*s < 0.02, suggesting overall lower ED symptom severity at the start of their treatment. There were no admission differences in the frequency of self-reported compensatory behaviors and objective binge episodes between genders within any ED group, all |*t*|s < 1.7, all *p*s > 0.10.

At end-of-treatment, self-reported symptom severity improved for men with AN in terms of Eating Concerns, *F*(1, 26) = 5.59, *p* = 0.03, *η^2^* = 0.18, and for men with BED in terms of Eating and Weight Concerns, *F*(1, 100) = 5.00, *p* = 0.03, *η^2^* = 0.05 and *F*(1, 100) = 5.66, *p* = 0.02, *η^2^* = 0.05. The data showed further reductions for women with BED in terms of Restraint, *F*(1, 99) = 7.04, *p* = 0.01, *η^2^* = 0.07, and for women with BN in terms of eating episodes, *F*(1, 11) = 17.41, *p* < 0.001, *η^2^* = 0.61, binge days, *F*(1, 12) = 9.91, *p* = 0.01, *η^2^* = 0.45, and self-induced vomiting, *F*(1, 12) = 5.21, *p* = 0.04, *η^2^* = 0.30. However, it must be noted that these changes could be caused by the clinic’s regulations prohibiting restrictive eating and purging behaviors. Other changes were not significant, all *F*s < 3.70, all *p*s > 0.06. Changes in EDE-Q scores from admission to end-of-treatment, controlled for admission levels and treatment duration differences, remained largely comparable between gender groups for patients with AN, BN, BED, and EDNOS, with two exceptions: men with AN showed a more pronounced decrease in Shape Concerns than women with AN, *F*(1, 57) = 4.87, *p* = 0.03, *η^2^* = 0.08, and men with BED showed a more pronounced decrease in the frequency of eating episodes than women with BED, *F*(1, 199) = 4.25, *p* = 0.04, *η^2^* = 0.02, though the clinic’s regulations on eating behavior again limit interpreting the latter difference. No other significant differences emerged, all *F*s < 3.6, all *p*s > 0.08.

We further examined FBeK body perception and body image scores (see Appendix A Table A1). At admission, men with BED scored higher in Attractiveness/Self-confidence compared to women with BED, *t*(191) = 2.34, *p* = 0.02, *d* = 0.31, 95% CI (0.05, 0.57), lower on Accentuation, *t*(224) = −3.98, *p* < 0.001, *d* = −0.52, 95% CI (−0.78, −0.26), and lower on Insecurities, *t*(214) = −2.33, *p* = 0.02, *d* = −0.31, 95% CI (−0.56, −0.05). Other admission comparisons were not significant, all |*t*|s < 1.22, all *p*s > 0.26. At end-of-treatment, only men with BED improved in Attractiveness/Self-confidence, *F*(1, 96) = 5.37, *p* = 0.02, *η^2^* = 0.05; other *F*s < 1.7, *p*s > 0.20. With the exception of men and women with BN, and women with EDNOS, Accentuation increased for all other patients, *F*s > 12.77, *p*s < 0.001. Insecurities decreased in women with BN, *F*(1, 11) = 9.00, *p* = 0.01, *η^2^* = 0.45, and in men with BED, *F*(1, 96) = 8.20, *p* = 0.01, *η^2^* = 0.08; other *F*s 3.96, *p*s > 0.09. Physical/Sexual Discomfort decreased for men with AN, *F*(1, 28) = 6.41, *p* = 0.02, *η^2^* = 0.19, and men with BN, *F*(1, 12) = 5.88, *p* = 0.03, *η^2^* = 0.33; other *F*s < 3.09, *p*s > 0.09. Changes in FBeK scores from admission to end-of-treatment, controlled for admission (baseline) levels and treatment duration differences, remained comparable between gender groups for patients with AN, BN, BED, and EDNOS, all *F*s < 2.37, *p*s > 0.13.

### 3.4. General Psychopathology Outcomes

Appendix A Table A1 further includes the scores of SCL-27-plus outcomes. At admission, men with AN reported fewer sociophobic symptoms and pain than women with AN, *t*(85) = −2.51, *p* = 0.01, *d* = −0.54, 95% CI (−0.97, −0.11) and *t*(85) = −2.17, *p* = 0.03, *d* = −0.47, 95% CI (−0.89, −0.04); men with BED reported fewer vegetative symptoms, *t*(236) = −3.16, *p* < 0.001, *d* = −0.41, 95% CI (−0.67, −0.15), fewer sociophobic symptoms, *t*(236) = −3.07, *p* < 0.001, *d* = −0.40, 95% CI (−0.65, −0.14), less pain, *t*(235) = −2.00, *p* = 0.05, *d* = −0.26, 95% CI (−0.52, −0.01), and presented with a lower global severity index than women with BED, *t*(237) = −2.81, *p* = 0.01, *d* = −0.36, 95% CI (−0.62, −0.11). Other admission comparisons, including between men and women with BN and EDNOS, were not significant, all |*t*|s < 1.9, all *p*s > 0.07.

At end-of-treatment, sociophobic symptoms decreased in men with AN, *F*(1, 26) = 5.60, *p* = 0.03, *η^2^* = 0.18, pain decreased in both men and women with AN, *F*(1, 26) = 5.46, *p* = 0.03, *η^2^* = 0.17 and *F*(1, 30) = 5.83, *p* = 0.02, *η^2^* = 0.16, and women with AN had a lower global severity index, *F*(1, 30) = 4.35, *p* = 0.05, *η^2^* = 0.13. Reduced pain and long-term depression scores were found among men with EDNOS, *F*(1, 6) = 6.68, *p* = 0.04, *η^2^* = 0.53 and *F*(1, 5) = 10.40, *p* = 0.02, *η^2^* = 0.68, and reduced depressive symptoms were found for women with EDNOS, *F*(1, 5) = 7.14, *p* = 0.04, *η^2^* = 0.59. Other differences, including for patients with BN and BED, were not significant, all *F*s < 3.97, all *p*s > 0.06. Compared to their female counterparts, men with AN and men with EDNOS showed more pronounced reductions in vegetative symptoms from admission to end-of-treatment, *F*(1, 58) = 5.10, *p* = 0.03, *η^2^* = 0.08 and *F*(1, 13) = 5.35, *p* = 0.04, *η^2^* = 0.29. Because women with BED reported more lifetime depression episodes at end-of-treatment, men with BED also showed relative improvements in lifetime depression in comparison, *F*(1, 189) = 8.77, *p* < 0.001, *η^2^* = 0.04. Other gender differences were not significant, all *F*s < 3.9, *p*s > 0.06.

Finally, we examined BDI outcomes (see Appendix A Table A1). Men with BED presented with lower severity of depression at admission compared to women with BED, *t*(225)= −2.25, *p* = 0.013 *d* = −0.29, 95% CI (−0.55, −0.04); other admission differences were not significant, all |*t*|s < 0.9, all *p*s > 0.38. There were, overall, no significant improvements in BDI scores from admission to end-of-treatment across groups, all *F*s < 1.5, all *p*s > 0.24, and no gender differences for the comparison of change scores, all *F*s < 3.8, all *p*s > 0.07.

### 3.5. Evidence Strength

**Figure 2:**
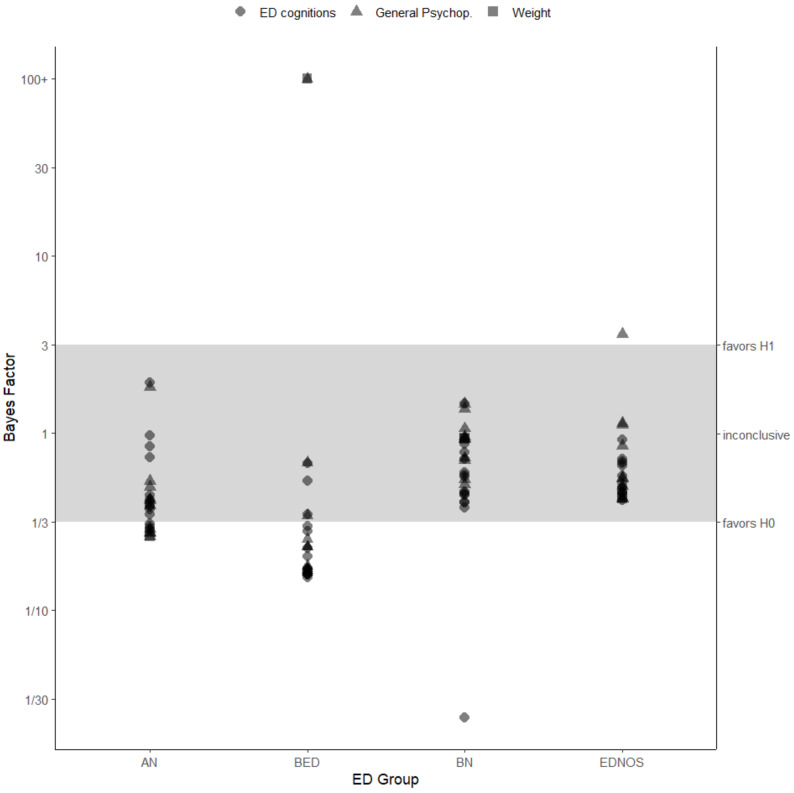
Inclusion Bayes Factor (BF_incl_) as an index of evidence strength stratified by ED group and outcome measure category. BF_incl_ ≥ 3 is considered as evidence for H1 (gender modulates the outcome) and, vice versa, a BF_incl_ ≤ 1/3 indicates evidence in favor of the H0 (gender does not modulate the outcome). ED = Eating Disorder, AN = Anorexia Nervosa, BN = Bulimia Nervosa, BED = Binge Eating Disorder, EDNOS = Eating Disorder Not Otherwise Specified.

Figure 2 plots the inclusion Bayes Factors obtained from Bayesian ANCOVA on gender differences in ED treatment outcomes, separated by ED group (see also Appendix A Table A2). Except for weight outcomes and SCL-27-plus lifetime depression, which provide strong evidence for H1 (i.e., that gender modulates these outcomes), most outcomes compared between men and women with BED (19 of 26) favor H0 (gender parity). In other words, for most outcomes in patients with BED, the data are at least three times more likely under statistical models that do not include gender than under models with that predictor. For patients with AN, about a third of outcomes (8 of 26) provide at least moderate support for H0, with evidence for the remaining outcomes remaining anecdotal, despite nominally supporting H0. Due to their smaller sample sizes, most outcomes comparisons for patients with BN and EDNOS remain within the anecdotal range, though generally favoring H0 over H1.

## 4. Discussion

EDs increasingly emerge as a health risk in men [15], yet men remain underrepresented in ED research and interventional trials [18]. Addressing concerns that men’s underrepresentation may have facilitated the development of women-centered ED treatments that result in suboptimal outcomes for men [26], we systematically compared immediate treatment outcomes between age-, diagnosis-, and length-of-treatment-matched samples of men and women with AN, BN, BED, and EDNOS, treated at the same clinic during the same time period, and using the same measurements. Compared to their female counterparts, men with AN showed improved weight gains during treatment and improved in ED-related cognitions and general psychopathology. Likewise, men with BED showed improved weight loss during treatment compared to women with BED, with ED-related cognitions and general psychopathology outcomes remaining comparable. For BN and EDNOS, weight, ED-related cognitions, and general psychopathology outcomes remained largely comparable between men and women.

The present findings add to an emerging yet, overall, still sparse body of studies that systematically compare treatment outcomes between men and women with EDs. Consistent with adolescent [45,46] and adult AN samples [51], we observed improved weight gains in men compared to women with AN throughout treatment. Although there were no significant gender differences when comparing age- and gender-standardized BMIs at admission to end-of-treatment, men showed more pronounced weight increases after the first week of treatment. However, the mechanisms responsible for these improved weight gains remain elusive. Moreover, and in contrast to Strobel et al. [51], who observed more pronounced reductions in ED-related cognitions in men with AN long-term, immediate reductions in ED-related cognitions at end-of-treatment remained comparable between men and women with AN in our sample. The increased weight change in the absence of gender differences in ED-related cognitions might suggest higher levels of therapy adherence (i.e., higher capacity for men to implement behavioral change despite the presence of ED-related cognitions) as a possible explanation for gender differences in weight gains. As noted above, however, the possibility remains that traditional ED-specific assessments may simply not have captured improved ED-related cognition outcomes due to traditional measures failing to account for men-associated symptomatology [19]. Further research is needed on the underlying mechanisms of improved weight gains in men with AN.

We observed a complementary pattern of increased weight loss in men with BED throughout treatment, consistent with gender differences found in clinical trial data [50]. The similarities further extend toward ED-specific psychopathology: men with BED in our study presented with less severe ED-related cognitions and a more positive body image than women with BED, although men showed similar improvements to women with BED due to their treatment. Again, the reasons for pronounced weight reductions with simultaneous parity in ED-related cognition outcomes remain elusive, as we cannot exclude that traditional ED-specific assessments may be less sensitive toward capturing men-specific ED psychopathology. Men with BED may show pronounced weight reductions due to higher levels of energy expenditure [76], although increased weight reductions could also reflect differences in therapy adherence. Given the paucity of studies on gender differences, especially for treatment outcomes in BED, further substantiation of these observations and their long-term consequences is needed. Shedding light onto the reasons that could be responsible for such differences between men and women may advance our understanding even further for the more tenacious course of weight gain in women’s AN and support the design of corresponding interventions.

Gender differences were further examined for patients with BN and EDNOS. Like Strobel et al. [51], but unlike Fernández-Aranda et al. [49], we did not observe gender differences between men and women treated for BN. However, given the limited number of patients involved in the comparisons, we caution against strong interpretations. Similarly, we caution against interpreting the absence of gender differences among patients with EDNOS, although the overall pattern of findings favors gender parity.

Thus far, the role of gender and other diversity aspects remain poorly explored in ED treatment settings [13], raising the question of whether men and women with EDs should be treated differently. The present findings suggest two possible implications: First, evidence for gender parity in levels of ED-related and general psychopathology suggests that current diagnostics provide adequate tools for ED assessment across gender groups. At the same time, until future research has thoroughly examined, established or refuted the validity of these tools for cross-gender ED assessments, presumptions and stereotypical expectations about gendered ED presentation should not preclude men from receiving comprehensive diagnostics. Second, observed gender differences in the speed and magnitude of weight changes for AN and BED groups suggest that therapists should employ different criteria when evaluating ED treatment outcomes for men and women. However, further large-scale and controlled comparisons are required in order to develop more specific recommendations.

### Strengths and Limitations

To the best of our knowledge, this is one of the first large-scale pre-/post-treatment comparisons of gender differences in ED treatment outcomes involving diagnosis-, age-, and length-of-treatment-matched men and women with AN, BN, BED, and EDNOS. All patients were treated in the same clinic during the same timeframe and completed the same standardized ED-specific and general psychopathology measures. Although the study design precluded strict control of treatment application, the resulting naturalistic setting allows for a more direct evaluation of ED treatment effectiveness across men and women.

Interpreting the current findings is subject to limitations. With data collected exclusively at an EDs specialty clinic, evidence for gender parity could be limited to the more severe ED cases admitted to inpatient treatment, or to treatment settings with high levels of expertise and experience. Moreover, given possible deviations from treatment protocols under naturalistic conditions and the retrospective nature of the study, we cannot exclude that therapists may have compensated for specific men’s needs that are not addressed during standard treatment. We also only report on immediate treatment outcomes, raising the question of whether long-term outcomes would remain comparable among these patients.

Moreover, as mentioned above, diagnostic criteria and ED-specific assessment tools were developed primarily based on EDs in women’s samples, questioning whether their use may have promoted phenotypical homogeneity among the men and women with EDs that were included in our sample. For example, we observed similar levels in weight and shape concerns between gender groups at admission, while previous research shows that men with EDs are often less concerned with thinness [22,23,24] and may seek to increase body mass and muscularity instead [25,26]. “Gold standard” measurement tools of ED psychopathology such as the EDE-Q used within this study [77] do not currently distinguish between drives for muscularity and thinness as non-exclusive causes of shape and weight concerns, leaving the possibility that gender differences in ED psychopathology could have been present without being detected. The extent to which men-associated body image concerns may have or may not have been adequately addressed thus cannot be answered based on the present data.

Finally, the current data provide only limited insights into gender differences concerning risk factors and antecedents of ED development. Still, the observed similarities in ED and general psychopathology at admission may suggest shared risk factors across genders, though their particularities might vary. For example, engaging in sharing technologically enhanced (“filtered”) images and comparisons on social networking sites has been linked to body dissatisfaction and eating disorder risk in adolescent women [78], and similar patterns have been observed concerning idealized representations of muscularity in men [79]. However, similar to other aspects of diversity in ED research, gender differences in ED development remain poorly explored [13]. Therefore, further research on ED presentation, assessment, and treatment in men is warranted.

## 5. Conclusions

Gender differences in ED treatment outcomes remain under-explored. Our data provide at least moderate support for gender parity and against gender differences among ED and general psychopathology outcomes in BED and AN treatment, with weight outcomes favoring men over women. Further research on underlying mechanisms and gender differences among men-associated ED outcomes is needed.

## Figures and Tables

**Table 1:** Patient and treatment characteristics.

ED Group *	Gender	*n*	Age			DOT		
			M	SD	*p*	M	SD	*p*
AN	men	47	24.57	8.54	0.51	43.53	22.69	0.44
	women	47	22.94	9.64		46.34	22.61	
BN	men	18	33.39	10.12	0.23	44.06	17.10	0.48
	women	18	28.50	9.00		48.17	14.26	
BED	men	125	34.78	13.90	0.86	45.37	15.31	0.10
	women	125	34.50	12.48		49.02	14.87	
EDNOS	men	10	30.70	14.20	0.99	49.30	25.71	0.47
	women	10	30.80	12.02		43.70	13.01	

* Means (Ms) and standard deviations (SDs) for patients’ age and days of treatment (DOT), stratified by ED diagnosis and gender. Bonferroni-corrected *p*-values indicate gender differences based on 2 (gender) × 4 (ED group) ANOVAs. ED = Eating Disorder, AN = Anorexia nervosa, BN = Bulimia nervosa, BED = Binge Eating Disorder, EDNOS = Eating Disorder Not Otherwise Specified.

**Table 2:** LME model inferential statistics for change in weight outcomes.

Predictor *	*χ^2^*	*df*	*p*
(Intercept)	21.06	1	**0.000**
DOT	482.95	1	**0.000**
ED Group	10.51	3	**0.01**
Gender	2.18	1	0.14
DOT × ED Group	557.67	3	**0.000**
DOT × Gender	26.49	1	**0.000**
ED Group × Gender	4.92	3	0.18
DOT × ED Group × Gender	83.46	3	**0.000**

* DOT = day of treatment, LME = linear mixed-effects. Bold values indicate *p* < 0.05. *χ^2^*and *df* are statistical elements.

## Data Availability

The data presented in this study are available upon reasonable request from the corresponding author.

## Appendix A

Table A1 and Table A2 present additional descriptive and inferential statistics for ED outcomes analyses, respectively. Table A3 includes additional results for LME modelling of weight outcomes.

**Table A1:** Outcome measures descriptive statistics.

ED Group:	Men									Women								
Variables *	Admission			End-Of-Treatment			Change EOT-Ad.			Admission			End-Of-Treatment			Change EOT-Ad.		
	*n*	M	SD	*n*	M	SD	*n*	M	SD	*n*	M	SD	*n*	M	SD	*n*	M	SD
**AN**																		
Weight																		
BMI	47	17.46	2.97	47	17.94	2.39	47	0.49	1.37	47	17.36	2.45	47	17.68	2.30	47	0.31	1.18
zBMI	47	−2.63	1.62	47	−2.26	1.23	47	0.38	0.87	47	−2.15	1.52	47	−1.95	1.44	47	0.20	0.73
EDEQ																		
total	46	4.12	1.44	31	2.54	0.85	30	−1.75	1.22	44	4.34	1.49	34	3.05	1.23	32	−1.49	1.07
Restraint	46	3.84	1.92	31	1.81	0.76	30	−2.24	1.50	44	4.21	2.17	34	2.16	1.20	32	−2.50	1.75
EatingConcern	45	3.69	1.57	31	2.10	0.85	29	−1.64	1.37	44	3.86	1.31	34	2.58	1.26	32	−1.30	1.04
WeightsConcern	45	4.25	1.44	31	2.82	1.05	29	−1.54	1.49	44	4.35	1.67	34	3.26	1.53	32	−1.25	1.28
ShapeConcern	45	4.73	1.59	31	3.43	1.33	29	−1.58	1.43	44	4.95	1.53	34	4.22	1.56	32	−0.93	1.14
f (eat. episode)	45	4.87	8.55	31	1.68	5.43	29	−2.45	9.35	44	4.14	8.01	34	3.88	11.33	32	−0.28	13.93
f (loss control)	44	3.80	7.25	31	0.52	0.93	29	−3.14	6.06	43	4.60	8.43	34	0.15	0.44	31	−3.71	7.38
f (binge days)	44	3.95	7.46	31	1.03	3.63	29	−3.10	4.45	43	4.84	8.80	34	0.29	0.80	31	−3.68	6.80
f (purge)	44	5.57	16.69	31	0.42	1.50	29	−6.17	19.02	44	3.14	7.61	34	0.53	2.12	32	−1.53	4.45
f (lax.)	44	0.18	0.69	31	0.00	0.00	29	−0.28	0.84	44	0.95	3.67	34	0.06	0.34	32	−0.56	2.50
f (exer.)	43	7.42	10.05	31	3.06	6.24	28	−3.71	8.92	44	8.39	10.27	34	2.06	5.14	32	−7.19	10.91
FBeK																		
Attract.	46	6.30	9.25	32	12.09	14.44	31	6.74	11.56	45	6.38	10.80	34	10.94	14.38	33	4.18	14.47
Accent.	46	75.87	27.25	32	81.81	23.25	31	6.45	32.57	45	76.02	26.64	34	88.15	17.11	33	5.33	21.38
Worry	46	80.48	24.94	32	76.53	26.53	31	−7.58	25.21	45	78.07	26.86	34	79.32	21.87	33	0.18	25.83
Sex.Discomfort	46	81.37	23.52	32	82.06	21.44	31	−1.39	19.70	45	79.98	22.70	34	78.59	26.53	33	−3.33	24.51
SCL-27-plus																		
Dep	43	2.18	0.93	31	1.40	0.95	29	−0.65	0.89	44	2.27	0.94	35	1.66	1.15	33	−0.65	0.82
Veg	43	1.68	0.86	31	1.23	0.75	29	−0.44	0.59	44	1.93	0.96	35	1.74	0.97	33	−0.18	0.54
Ago	43	1.03	0.96	31	0.84	0.97	29	−0.17	0.57	44	1.13	0.93	35	1.05	0.87	33	−0.11	0.71
Sop	43	2.08	1.06	31	2.00	0.99	29	−0.11	0.73	44	2.60	0.89	35	2.61	0.93	33	−0.02	0.54
Pain	43	1.45	0.82	31	1.24	0.79	29	−0.19	0.63	44	1.85	0.87	35	1.66	0.84	33	−0.16	0.54
GSI	43	1.68	0.63	31	1.34	0.65	29	−0.31	0.47	44	1.96	0.71	35	1.74	0.76	33	−0.22	0.37
LTDep	40	0.58	0.36	27	0.66	0.31	24	0.12	0.39	44	0.60	0.40	35	0.74	0.35	33	0.14	0.44
f (suic. thoughts)	45	1.11	2.52	31	0.71	1.35	29	−0.48	2.40	46	9.15	53.72	35	0.83	3.38	34	−0.21	1.37
f (suic. attempts)	46	0.09	0.35	31	0.23	0.67	30	0.10	0.55	46	0.61	2.04	35	0.37	1.70	34	−0.09	0.57
BDI																		
total score	46	25.04	12.83	32	13.66	10.67	31	−11.77	11.15	46	26.04	13.23	35	17.06	11.88	34	−8.29	12.98
**BN**																		
Weight																		
BMI	18	28.63	9.44	18	27.05	8.44	18	−1.59	1.52	18	25.35	6.30	18	24.59	5.85	18	−0.77	1.09
zBMI	18	0.35	1.72	18	0.07	1.58	18	−0.28	0.32	18	0.29	1.16	18	0.14	1.14	18	−0.16	0.19
EDEQ																		
total	18	5.49	1.18	15	3.54	1.81	15	−1.84	1.40	18	5.59	0.89	15	3.24	1.45	15	−2.46	1.59
Restraint	18	5.29	1.87	15	2.74	1.57	15	−2.28	1.66	18	5.28	1.70	15	2.40	1.20	15	−3.32	1.61
EatingConcern	18	4.80	1.19	15	3.01	1.94	15	−1.84	1.83	18	4.85	1.18	15	2.68	1.37	15	−2.09	1.71
WeightsConcern	18	5.72	1.41	15	4.00	2.18	15	−1.56	1.73	18	5.96	1.08	15	3.69	1.89	15	−2.38	1.90
ShapeConcern	18	6.14	1.31	15	4.40	2.08	15	−1.67	1.68	18	6.28	0.86	15	4.18	1.75	15	−2.07	1.84
f (eat. episode)	18	19.11	11.93	15	3.20	7.86	15	−15.67	13.22	17	13.00	9.38	15	0.80	1.26	14	−11.93	8.77
f (loss control)	18	18.39	11.10	15	2.00	4.42	15	−16.20	11.43	18	13.17	8.95	15	2.20	5.09	15	−9.20	6.26
f (binge days)	18	18.00	10.20	15	1.80	3.99	15	−15.87	10.25	18	14.00	9.52	15	0.87	1.30	15	−12.33	8.60
f (purge)	18	13.94	11.88	15	7.87	16.67	15	−5.93	19.03	18	13.17	12.46	15	0.80	1.66	15	−13.13	11.80
f (lax.)	18	4.39	8.81	15	0.07	0.26	15	−3.87	8.39	18	2.56	5.17	15	0.00	0.00	15	−2.27	5.01
f (exer.)	18	7.22	11.03	15	6.53	13.54	15	−0.20	12.07	18	11.28	14.38	15	1.07	1.83	15	−12.47	14.81
FBeK																		
Attract.	17	7.65	19.67	16	15.31	25.29	15	7.80	19.93	17	2.41	1.77	14	19.57	24.78	14	17.36	24.64
Accent.	17	78.53	24.62	16	80.63	23.09	15	3.87	12.64	17	87.53	18.02	14	90.14	16.03	14	5.00	9.89
Worry	17	82.35	23.98	16	73.69	33.64	15	−8.60	18.63	17	87.76	16.72	14	83.14	14.19	14	−3.36	12.68
Sex.Discomfort	17	91.53	14.50	16	85.69	21.96	15	−5.67	10.40	17	87.00	14.46	14	81.36	21.39	14	−8.64	18.86
SCL-27-plus																		
Dep	18	2.42	1.24	15	1.76	1.47	15	−0.67	1.07	18	2.28	0.83	15	1.23	0.97	15	−1.13	1.02
Veg	18	1.80	1.06	15	1.48	1.01	15	−0.19	0.36	18	2.27	0.79	15	1.50	0.80	15	−0.71	0.88
Ago	18	1.57	1.31	15	1.35	1.32	15	−0.20	0.99	18	1.31	0.99	14	0.95	0.91	14	−0.45	0.66
Sop	18	2.78	1.26	15	2.41	1.43	15	−0.32	0.88	18	2.92	0.97	15	2.25	0.97	15	−0.77	0.87
Pain	18	1.90	1.03	15	1.71	1.01	15	−0.01	0.55	18	2.06	0.76	15	1.49	0.62	15	−0.46	0.67
GSI	18	2.09	0.92	15	1.74	1.11	15	−0.28	0.58	18	2.17	0.53	15	1.50	0.71	15	−0.67	0.70
LTDep	18	0.82	0.30	14	0.79	0.32	14	−0.11	0.23	18	0.70	0.37	13	0.82	0.36	13	0.08	0.28
f (suic. thoughts)	15	3.07	5.04	15	1.60	2.16	13	−2.15	5.81	18	0.72	0.96	15	0.53	0.83	15	−0.33	0.98
f (suic. attempts)	18	1.00	2.00	15	1.93	3.56	15	0.73	3.17	18	0.17	0.38	15	0.33	0.62	15	0.13	0.35
BDI																		
total score	18	31.06	15.09	15	21.87	17.67	15	−8.40	7.88	18	27.33	9.62	14	12.57	9.09	14	−15.43	11.24
**BED**																		
Weight																		
BMI	125	47.52	9.04	125	43.95	8.16	125	−3.57	1.74	125	42.82	9.06	125	40.54	8.54	125	−2.29	1.29
zBMI	125	3.23	0.85	125	2.92	0.86	125	−0.31	0.15	125	2.28	0.69	125	2.13	0.72	125	−0.15	0.11
EDEQ																		
total	118	4.05	0.92	108	2.82	1.00	103	−1.26	1.03	115	4.50	0.97	105	2.99	1.06	102	−1.53	1.15
Restraint	118	2.36	1.24	108	1.96	1.14	103	−0.41	1.50	115	2.79	1.47	105	1.92	1.00	102	−0.90	1.72
EatingConcern	118	3.68	1.52	108	2.08	1.03	103	−1.67	1.45	115	4.13	1.38	105	2.32	1.10	102	−1.84	1.46
WeightsConcern	118	4.94	0.98	108	3.62	1.23	103	−1.34	1.26	115	5.42	1.09	105	3.77	1.36	102	−1.63	1.41
ShapeConcern	118	5.22	1.05	108	3.60	1.42	103	−1.62	1.37	115	5.67	1.01	105	3.95	1.48	102	−1.74	1.44
f (eat. episode)	117	14.35	11.98	108	1.26	2.95	102	−13.58	12.66	114	14.10	9.00	105	0.68	1.53	101	−13.24	8.49
f (loss control)	116	12.22	11.84	108	1.04	2.37	101	−11.55	12.42	111	12.06	9.69	104	1.13	4.03	97	−10.93	9.98
f (binge days)	117	11.68	8.79	108	0.87	1.98	102	−11.25	8.95	112	12.63	9.60	105	0.91	3.61	99	−11.83	9.31
f (purge)	114	0.18	0.88	107	0.14	0.78	99	−0.01	0.61	115	0.22	1.07	104	0.08	0.53	101	0.00	0.60
f (lax.)	115	0.15	1.21	108	0.04	0.23	100	0.01	0.10	114	0.20	1.88	104	0.01	0.10	101	−0.22	2.00
f (exer.)	116	1.61	4.62	108	1.29	2.69	101	−0.26	4.56	115	1.09	3.69	105	1.98	4.69	102	0.78	5.72
FBeK																		
Attract.	116	3.79	4.25	104	10.62	14.07	99	7.02	13.69	117	2.71	2.61	109	11.71	18.49	106	9.25	17.76
Accent.	116	61.78	28.19	104	69.74	27.24	99	8.04	26.04	117	75.41	23.87	109	80.52	22.31	106	4.78	22.78
Worry	116	81.91	21.80	104	72.36	25.85	99	−9.20	23.94	117	87.80	16.47	109	77.84	25.31	106	−9.70	25.33
Sex.Discomfort	116	83.81	19.92	104	73.33	25.59	99	−10.74	23.81	117	85.64	20.28	109	79.50	24.13	106	−5.96	22.48
SCL−27-plus																		
Dep	118	1.77	1.02	107	1.16	1.06	101	−0.66	0.96	121	1.83	0.85	110	1.22	0.92	107	−0.60	0.92
Veg	118	1.25	0.72	107	1.03	0.77	101	−0.28	0.60	120	1.56	0.81	111	1.29	0.83	107	−0.21	0.68
Ago	118	0.89	0.86	107	0.69	0.76	101	−0.20	0.56	119	1.05	0.91	111	0.90	0.90	106	−0.08	0.63
Sop	118	2.01	1.12	107	1.68	1.13	101	−0.36	0.88	120	2.43	1.02	111	2.05	1.05	107	−0.40	0.84
Pain	118	1.93	0.78	107	1.68	0.77	101	−0.27	0.59	119	2.14	0.83	111	1.84	0.80	106	−0.27	0.63
GSI	118	1.57	0.68	107	1.25	0.70	101	−0.35	0.50	121	1.81	0.63	111	1.46	0.69	108	−0.31	0.53
LTDep	112	0.58	0.36	94	0.56	0.38	86	−0.04	0.33	120	0.66	0.30	110	0.74	0.32	107	0.07	0.26
f (suic. thoughts)	118	1.81	4.72	107	3.02	11.89	101	0.71	11.91	120	4.90	28.90	111	4.70	18.60	109	2.49	14.26
f (suic. attempts)	119	0.15	0.46	107	0.20	0.57	102	0.04	0.24	121	0.21	0.61	111	0.22	0.55	108	0.02	0.24
BDI																		
total score	118	22.15	10.72	107	13.61	10.59	101	−8.90	9.94	122	24.99	8.67	112	14.60	10.39	110	−10.46	10.13
**EDNOS**																		
Weight																		
BMI	10	29.62	14.14	10	27.29	9.96	10	−2.33	5.77	10	25.48	8.57	10	24.81	7.54	10	−0.67	1.27
zBMI	10	0.13	2.61	10	0.07	2.16	10	−0.07	0.58	10	0.10	1.33	10	0.01	1.28	10	−0.09	0.20
EDEQ																		
total	10	3.48	1.23	9	2.49	1.05	9	−0.97	0.94	9	3.83	1.62	8	2.26	1.19	8	−1.36	1.42
Restraint	10	2.14	1.46	9	1.29	0.47	9	−0.98	1.12	9	3.08	1.73	8	1.70	1.05	8	−1.34	1.85
EatingConcern	10	3.10	1.07	9	2.20	1.46	9	−0.97	1.24	9	3.38	1.65	8	2.03	1.16	8	−1.08	1.58
WeightsConcern	10	4.18	1.66	9	3.22	1.50	9	−0.80	1.12	9	4.19	1.81	8	2.48	1.14	8	−1.44	1.40
ShapeConcern	10	4.50	1.67	9	3.26	1.40	9	−1.11	1.15	9	4.65	2.14	8	2.82	1.75	8	−1.58	1.90
f (eat. episode)	10	5.20	8.79	8	2.50	3.46	8	−0.38	5.32	8	6.13	11.49	8	0.50	1.41	7	−3.43	9.07
f (loss control)	10	2.90	3.98	8	0.88	1.13	8	−1.38	3.34	8	7.50	10.36	8	0.63	1.19	7	−4.86	9.03
f (binge days)	10	2.50	3.21	8	1.50	2.73	8	−0.88	3.68	8	6.50	12.08	8	0.38	1.06	7	−3.00	7.94
f (purge)	10	0.80	2.53	8	0.38	1.06	8	−0.63	1.77	9	3.11	9.33	8	0.00	0.00	8	−3.50	9.90
f (lax.)	10	0.00	0.00	8	0.00	0.00	8	0.00	0.00	9	0.00	0.00	8	0.00	0.00	8	0.00	0.00
f (exer.)	10	2.50	7.91	8	3.88	9.80	8	0.75	14.21	9	5.78	11.48	8	0.88	2.10	8	−5.63	10.45
FBeK																		
Attract.	10	5.40	6.19	9	12.11	8.37	9	6.11	8.13	9	8.56	10.45	8	14.25	6.39	8	5.13	13.29
Accent.	10	86.70	14.27	9	88.33	11.66	9	2.11	20.23	9	83.67	19.75	8	89.50	13.79	8	7.88	15.08
Worry	10	86.50	19.61	9	86.78	15.63	9	0.11	8.19	9	80.67	20.69	8	70.13	35.04	8	−10.88	26.35
Sex.Discomfort	10	85.30	19.77	9	71.56	21.97	9	−12.33	20.89	9	74.89	25.55	8	65.00	30.92	8	−8.88	13.53
SCL−27-plus																		
Dep	10	1.94	0.57	9	1.40	1.07	9	−0.51	0.69	9	1.60	0.66	8	1.03	0.46	8	−0.55	0.45
Veg	10	1.58	0.71	9	1.13	0.81	9	−0.53	0.36	9	1.44	0.65	8	1.28	0.38	8	0.00	0.43
Ago	10	1.03	0.91	9	0.81	0.93	9	−0.25	0.28	9	0.75	0.64	8	0.72	0.84	8	−0.06	0.53
Sop	10	1.90	1.03	9	1.62	0.75	9	−0.44	0.70	9	1.82	1.41	8	1.73	1.22	8	−0.15	0.58
Pain	10	2.06	0.59	9	1.76	0.35	9	−0.24	0.48	9	1.76	0.86	8	1.58	0.57	8	−0.13	0.40
GSI	10	1.70	0.45	9	1.34	0.58	9	−0.40	0.33	9	1.48	0.58	8	1.27	0.52	8	−0.18	0.24
LTDep	10	0.69	0.32	8	0.58	0.38	8	−0.06	0.11	9	0.41	0.43	7	0.60	0.43	7	0.22	0.42
f (suic. thoughts)	10	0.70	1.34	9	0.67	1.41	9	−0.11	0.33	9	0.00	0.00	8	0.38	0.74	8	0.38	0.74
f (suic. attempts)	9	0.11	0.33	9	0.22	0.67	8	0.13	0.35	9	0.00	0.00	8	0.00	0.00	8	0.00	0.00
BDI																		
total score	10	23.00	10.01	9	12.44	10.74	9	−10.00	11.62	9	20.22	7.79	8	8.63	3.16	8	−11.75	6.39

* Means (M) and standard deviations (SDs) for outcome measures, stratified by ED diagnosis, gender, and time point. ED = Eating Disorder, EOT-Ad. = Differences between End-Of-Treatment and Admission values, AN = Anorexia Nervosa, BN = Bulimia Nervosa, BED = Binge Eating Disorder, EDNOS = Eating Disorder Not Otherwise Specified, BMI = Body Mass Index, zBMI = age- and gender-standardized BMI-Scores, EDEQ = Eating Disorder Examination Questionnaire, eat. = eating, lax. = laxatives, exer. = exercising, FBeK = Fragebogen zur Beurteilung des eigenen Körpers, Attract. = Physical Attractiveness and Self-confidence, Accent. = Accentuation of Physical Appearance, SCL-27-plus = Symptom Checklist SCL-27-plus, Dep = depressive symptoms, Veg = vegetative symptoms, Ago = agoraphobic symptoms, Sop = sociophobic symptoms, GSI = global symptom severity index, LTDep = lifetime assessment for depressive symptoms, suic. = suicide, BDI = Beck Depression Inventory.

**Table A2:** Outcome measures inferential statistics.

ED Group:	Admission Differences						Change from Adm. to EOT								Gender Differences of Change				
Variables *	Men vs. Women						Men				Women								
	*t*	*df*	*p*	*d*	*LL*	*UL*	*F*	*df*	*p*	*eta^2^*	*F*	*df*	*p*	*eta^2^*	*F*	*df*	*p*	*eta^2^*	*BFincl*
**AN**																			
Weight																			
BMI	0.17	92	0.865	0.04	−0.37	0.44	22.73	44	0.000	0.34	7.33	44	0.010	0.14	1.34	90	0.250	0.01	0.38
zBMI	−1.50	92	0.137	−0.31	−0.72	0.10	23.04	44	0.000	0.34	9.18	44	0.000	0.17	0.38	90	0.540	0.00	0.26
EDEQ																			
total	−0.71	88	0.477	−0.15	−0.56	0.264	1.96	27	0.170	0.07	1.42	29	0.240	0.05	2.58	58	0.110	0.04	0.73
Restraint	−0.87	88	0.389	−0.18	−0.60	0.23	0.48	27	0.490	0.02	0.25	29	0.620	0.01	0.32	58	0.570	0.01	0.29
EatingConcern	−0.53	87	0.595	−0.11	−0.53	0.30	5.59	26	0.030	0.18	0.50	29	0.480	0.02	2.85	57	0.100	0.05	0.84
WeightsConcern	−0.32	87	0.750	−0.07	−0.48	0.35	3.04	26	0.090	0.10	1.44	29	0.240	0.05	1.12	57	0.290	0.02	0.41
ShapeConcern	−0.68	87	0.500	−0.14	−0.56	0.27	0.00	26	0.950	0.00	0.95	29	0.340	0.03	4.87	57	0.030	0.08	1.93
f (eat. episode)	0.42	87	0.679	0.09	−0.33	0.50	0.26	26	0.610	0.01	0.00	29	0.990	0.00	0.98	57	0.330	0.02	0.39
f (loss control)	−0.48	85	0.632	−0.10	−0.52	0.32	0.16	26	0.700	0.01	0.73	28	0.400	0.03	3.31	56	0.070	0.06	0.97
f (binge days)	−0.51	85	0.615	−0.11	−0.53	0.31	0.85	26	0.360	0.03	0.00	28	0.970	0.00	1.39	56	0.240	0.02	0.45
f (purge)	0.88	86	0.381	0.19	−0.23	0.61	0.00	26	0.960	0.00	0.00	29	1.00	0.00	0.16	57	0.690	0.00	0.30
f (lax.)	−1.37	46	0.177	−0.29	−0.71	0.13	-	-	-	-	0.40	29	0.530	0.01	0.62	57	0.430	0.01	0.35
f (exer.)	−0.44	85	0.658	−0.10	−0.52	0.33	0.06	25	0.810	0.00	2.15	29	0.150	0.07	1.16	56	0.290	0.02	0.43
FBeK																			
Attract.	−0.04	89	0.972	−0.01	−0.42	0.40	0.22	28	0.650	0.01	0.00	30	0.950	0.00	0.50	60	0.480	0.01	0.31
Accent.	−0.03	89	0.979	−0.01	−0.42	0.41	19.66	28	0.000	0.41	14.94	30	0.000	0.33	1.09	60	0.300	0.02	0.39
Worry	0.44	89	0.658	0.09	−0.32	0.50	1.95	28	0.170	0.07	1.33	30	0.260	0.04	0.72	60	0.400	0.01	0.37
Sex.Discomfort	0.29	89	0.775	0.06	−0.35	0.47	6.41	28	0.020	0.19	3.08	30	0.090	0.09	0.28	60	0.600	0.00	0.29
SCL−27-plus																			
Dep	−0.46	85	0.649	−0.10	−0.52	0.32	0.82	26	0.370	0.03	0.44	30	0.510	0.01	0.02	58	0.880	0.00	0.26
Veg	−1.29	85	0.200	−0.28	−0.70	0.15	0.04	26	0.850	0.00	3.95	30	0.060	0.12	5.10	58	0.030	0.08	1.82
Ago	−0.48	85	0.632	−0.10	−0.52	0.32	0.09	26	0.760	0.00	2.17	30	0.150	0.07	0.17	58	0.680	0.00	0.27
Sop	−2.51	85	0.014	−0.54	−0.97	−0.11	5.60	26	0.030	0.18	2.84	30	0.100	0.09	1.49	58	0.230	0.03	0.42
Pain	−2.17	85	0.033	−0.47	−0.89	−0.04	5.46	26	0.030	0.17	5.83	30	0.020	0.16	1.14	58	0.290	0.02	0.39
GSI	−1.89	85	0.062	−0.41	−0.83	0.02	0.77	26	0.390	0.03	4.35	30	0.050	0.13	1.59	58	0.210	0.03	0.43
LTDep	−0.21	82	0.835	−0.05	−0.47	0.38	2.81	21	0.110	0.12	0.91	30	0.350	0.03	0.00	53	0.950	0.00	0.28
f (suic. thoughts)	−1.00	89	0.319	−0.21	−0.62	0.20	0.07	26	0.800	0.00	2.31	31	0.140	0.07	0.22	59	0.640	0.00	0.29
f (suic. attempts)	−1.71	48	0.094	−0.36	−0.77	0.06	2.79	27	0.110	0.09	2.27	31	0.140	0.07	1.27	60	0.260	0.02	0.50
BDI																			
total score	−0.37	90	0.714	−0.08	−0.49	0.33	0.03	28	0.860	0.00	0.58	31	0.450	0.02	1.68	61	0.200	0.03	0.54
**BN**																			
Weight																			
BMI	1.23	34	0.229	0.41	−0.26	1.07	4.48	15	0.050	0.23	7.76	15	0.010	0.34	3.10	32	0.090	0.09	0.94
zBMI	0.12	34	0.908	0.04	−0.62	0.69	0.08	15	0.780	0.01	0.21	15	0.660	0.01	3.20	32	0.080	0.09	0.93
EDEQ																			
total	−0.3	34	0.766	−0.1	−0.75	0.555	0.57	12	0.470	0.05	0.43	12	0.520	0.03	1.87	26	0.180	0.07	0.57
Restraint	0.02	34	0.985	0.01	−0.65	0.66	0.02	12	0.890	0.00	0.31	12	0.590	0.03	2.90	26	0.100	0.10	0.78
EatingConcern	−0.13	34	0.900	−0.04	−0.70	0.61	0.83	12	0.380	0.06	0.75	12	0.400	0.06	1.70	26	0.200	0.06	0.45
WeightsConcern	−0.56	34	0.581	−0.19	−0.84	0.47	0.42	12	0.530	0.03	0.08	12	0.780	0.01	1.62	26	0.210	0.06	0.60
ShapeConcern	−0.40	34	0.694	−0.13	−0.79	0.52	0.13	12	0.720	0.01	0.32	12	0.580	0.03	0.79	26	0.380	0.03	0.41
f (eat. episode)	1.68	33	0.103	0.57	−0.11	1.24	3.30	12	0.090	0.22	17.41	11	0.000	0.61	3.39	25	0.080	0.12	0.71
f (loss control)	1.55	34	0.130	0.52	−0.15	1.18	3.34	12	0.090	0.22	0.48	12	0.500	0.04	0.02	26	0.880	0.00	0.45
f (binge days)	1.22	34	0.232	0.41	−0.26	1.06	2.62	12	0.130	0.18	9.91	12	0.010	0.45	2.13	26	0.160	0.08	0.46
f (purge)	0.19	34	0.849	0.06	−0.59	0.72	0.06	12	0.820	0.00	5.21	12	0.040	0.30	2.18	26	0.150	0.08	0.87
f (lax.)	0.76	34	0.451	0.25	−0.40	0.91	3.00	12	0.110	0.20	0.00	12	1.00	0.00	2.56	26	0.120	0.09	0.03
f (exer.)	−0.95	34	0.349	−0.32	−0.97	0.34	0.85	12	0.370	0.07	1.99	12	0.180	0.14	2.55	26	0.120	0.09	1.48
FBeK																			
Attract.	1.09	16	0.290	0.38	−0.31	1.05	1.02	12	0.330	0.08	1.28	11	0.280	0.10	1.78	25	0.190	0.07	0.58
Accent.	−1.22	32	0.233	−0.42	−1.09	0.27	0.60	12	0.450	0.05	2.16	11	0.170	0.16	0.04	25	0.840	0.00	0.38
Worry	−0.76	32	0.451	−0.26	−0.94	0.42	1.59	12	0.230	0.12	9.00	11	0.010	0.45	0.51	25	0.480	0.02	0.47
Sex.Discomfort	0.91	32	0.369	0.31	−0.37	0.99	5.88	12	0.030	0.33	0.33	11	0.570	0.03	0.50	25	0.490	0.02	0.41
SCL−27-plus																			
Dep	0.41	34	0.684	0.14	−0.52	0.79	2.23	12	0.160	0.16	0.55	12	0.470	0.04	2.59	26	0.120	0.09	0.55
Veg	−1.49	34	0.145	−0.50	−1.16	0.17	0.01	12	0.930	0.00	0.10	12	0.750	0.01	2.38	26	0.130	0.08	1.06
Ago	0.68	34	0.499	0.23	−0.43	0.88	0.01	12	0.920	0.00	1.12	11	0.310	0.09	1.73	25	0.200	0.06	0.51
Sop	−0.39	34	0.702	−0.13	−0.78	0.53	0.30	12	0.600	0.02	0.05	12	0.820	0.00	2.50	26	0.130	0.09	0.73
Pain	−0.52	34	0.607	−0.17	−0.83	0.48	0.04	12	0.850	0.00	2.95	12	0.110	0.20	3.62	26	0.070	0.12	1.46
GSI	−0.29	27	0.776	−0.10	−0.75	0.56	0.89	12	0.360	0.07	0.45	12	0.520	0.04	2.65	26	0.120	0.09	0.93
LTDep	1.06	34	0.296	0.35	−0.31	1.01	0.20	11	0.670	0.02	0.02	10	0.890	0.00	0.73	23	0.400	0.03	0.96
f (suic. thoughts)	1.78	15	0.096	0.68	−0.03	1.38	0.00	10	0.980	0.00	0.01	12	0.910	0.00	3.80	24	0.060	0.14	0.93
f (suic. attempts)	1.74	18	0.099	0.58	−0.09	1.24	0.00	12	0.970	0.00	0.23	12	0.640	0.02	1.04	26	0.320	0.04	0.71
BDI																			
total score	0.88	34	0.384	0.29	−0.37	0.95	0.70	12	0.420	0.05	0.27	11	0.610	0.02	3.71	25	0.070	0.13	1.37
**BED**																			
Weight																			
BMI	4.10	248	0.000	0.52	0.27	0.77	30.94	122	0.000	0.20	8.44	122	0.000	0.06	43.50	246	0.000	0.15	4.E+07
zBMI	9.69	238	0.000	1.23	0.95	1.50	8.18	122	0.000	0.06	6.72	122	0.010	0.05	96.50	246	0.000	0.28	∞
EDEQ																			
total	−3.67	231	0.000	−0.48	−0.74	−0.22	3.36	100	0.070	0.03	0.43	99	0.520	0	0.11	201	0.740	0.00	0.16
Restraint	−2.42	231	0.016	−0.32	−0.58	−0.06	3.65	100	0.060	0.04	7.04	99	0.010	0.07	0.92	201	0.340	0.00	0.20
EatingConcern	−2.39	231	0.018	−0.31	−0.57	−0.05	5.00	100	0.030	0.05	1.31	99	0.250	0.01	0.39	201	0.530	0.00	0.22
WeightsConcern	−3.55	231	0.000	−0.47	−0.73	−0.21	5.66	100	0.020	0.05	0.51	99	0.480	0.01	0.12	201	0.730	0.00	0.16
ShapeConcern	−3.37	231	0.001	−0.44	−0.70	−0.18	1.65	100	0.200	0.02	0.34	99	0.560	0.00	0.08	201	0.780	0.00	0.17
f (eat. episode)	0.18	229	0.856	0.02	−0.23	0.28	0.05	99	0.830	0.00	3.61	98	0.060	0.04	4.25	199	0.040	0.02	0.68
f (loss control)	0.11	225	0.916	0.01	−0.25	0.27	3.13	98	0.080	0.03	3.09	94	0.080	0.03	0.05	194	0.820	0.00	0.16
f (binge days)	−0.79	227	0.431	−0.10	−0.36	0.16	0.38	99	0.540	0.00	0.17	96	0.680	0.00	0.35	197	0.550	0.00	0.17
f (purge)	−0.32	227	0.747	−0.04	−0.30	0.22	0.67	96	0.410	0.01	0.00	98	0.980	0.00	0.13	196	0.720	0.00	0.15
f (lax.)	−0.26	227	0.796	−0.03	−0.29	0.23	0.97	98	0.330	0.01	0.06	98	0.810	0.00	0.04	197	0.840	0.00	0.16
f (exer.)	0.95	229	0.341	0.13	−0.13	0.38	1.30	98	0.260	0.01	0.41	99	0.520	0.00	1.57	199	0.210	0.01	0.35
FBeK																			
Attract.	2.34	191	0.020	0.31	0.05	0.57	5.37	96	0.020	0.05	1.70	103	0.200	0.02	2.36	201	0.130	0.01	0.30
Accent.	−3.98	224	0.000	−0.52	−0.78	−0.26	13.95	96	0.000	0.13	12.77	103	0.000	0.11	1.14	201	0.290	0.01	0.28
Worry	−2.33	214	0.021	−0.31	−0.56	−0.05	8.20	96	0.010	0.08	0.85	103	0.360	0.01	0.13	201	0.710	0.00	0.17
Sex.Discomfort	−0.70	231	0.488	−0.09	−0.35	0.17	1.63	96	0.200	0.02	1.02	103	0.320	0.01	2.20	201	0.140	0.01	0.54
SCL−27-plus																			
Dep	−0.57	237	0.572	−0.07	−0.33	0.18	0.07	98	0.790	0.00	1.14	104	0.290	0.01	0.07	204	0.800	0.00	0.17
Veg	−3.16	236	0.002	−0.41	−0.67	−0.15	0.47	98	0.500	0.00	0.79	104	0.380	0.01	1.41	204	0.240	0.01	0.34
Ago	−1.34	235	0.181	−0.17	−0.43	0.08	0.08	98	0.780	0.00	0.08	103	0.780	0.00	3.02	203	0.080	0.01	0.68
Sop	−3.07	236	0.002	−0.40	−0.65	−0.14	0.09	98	0.770	0.00	0.38	104	0.540	0.00	0.31	204	0.580	0.00	0.17
Pain	−2.00	235	0.047	−0.26	−0.52	0.00	0.68	98	0.410	0.01	0.14	103	0.710	0.00	0.20	203	0.650	0.00	0.18
GSI	−2.81	237	0.005	−0.36	−0.62	−0.11	0.02	98	0.890	0.00	0.56	105	0.450	0.01	0.86	205	0.350	0.00	0.25
LTDep	−1.82	217	0.070	−0.24	−0.50	0.02	0.91	83	0.340	0.01	2.68	104	0.100	0.03	8.77	189	0.000	0.04	12.406
f (suic. thoughts)	−1.15	236	0.252	−0.15	−0.40	0.11	3.21	98	0.080	0.03	0.81	106	0.370	0.01	0.83	206	0.360	0.00	0.23
f (suic. attempts)	−0.91	238	0.363	−0.12	−0.37	0.14	0.83	99	0.360	0.01	3.07	105	0.080	0.03	1.13	206	0.290	0.01	0.23
BDI																			
total score	−2.25	225	0.025	−0.29	−0.55	−0.04	0.00	98	0.970	0.00	1.42	107	0.240	0.01	0.37	207	0.540	0.00	0.17
**EDNOS**																			
Weight																			
BMI	0.79	18	0.439	0.35	−0.54	1.23	9.83	7	0.020	0.58	12.28	7	0.010	0.64	0.00	16	0.970	0.00	0.43
zBMI	0.04	13	0.972	0.02	−0.86	0.89	4.49	7	0.070	0.39	0.00	7	0.990	0.00	0.31	16	0.590	0.02	0.43
EDEQ																			
total	−0.53	17	0.603	−0.24	−1.14	0.664	0.04	6	0.850	0.01	1.66	5	0.250	0.25	0.39	13	0.540	0.03	0.49
Restraint	−1.28	17	0.217	−0.59	−1.50	0.34	3.60	6	0.110	0.37	0.83	5	0.400	0.14	0.43	13	0.520	0.03	0.47
EatingConcern	−0.46	17	0.653	−0.21	−1.11	0.70	0.48	6	0.510	0.07	2.05	5	0.210	0.29	0.02	13	0.890	0.00	0.42
WeightsConcern	−0.02	17	0.986	−0.01	−0.91	0.89	0.00	6	0.950	0.00	1.38	5	0.290	0.22	1.63	13	0.220	0.11	0.71
ShapeConcern	−0.17	17	0.867	−0.08	−0.98	0.82	0.79	6	0.410	0.12	1.97	5	0.220	0.28	0.54	13	0.470	0.04	0.49
f (eat. episode)	−0.19	16	0.849	−0.09	−1.02	0.84	3.13	5	0.140	0.39	0.00	4	1.00	0.00	3.60	11	0.080	0.25	0.92
f (loss control)	−1.19	9	0.267	−0.62	−1.56	0.35	3.28	5	0.130	0.40	0.03	4	0.870	0.01	0.79	11	0.390	0.07	0.52
f (binge days)	−0.91	8	0.390	−0.48	−1.42	0.47	0.91	5	0.380	0.15	0.00	4	1.00	0.00	2.12	11	0.170	0.16	0.69
f (purge)	−0.76	17	0.461	−0.35	−1.25	0.57	-	-	-	-	0.00	5	1.00	0.00	1.85	12	0.200	0.13	0.66
f (lax.)	-	-	-	-	-	-	-	-	-	-	-	-	-	-	-	-	-	-	-
f (exer.)	−0.73	17	0.474	−0.34	−1.24	0.576	1.13	5	0.340	0.18	1.03	5	0.360	0.17	1.02	12	0.330	0.08	0.58
FBeK																			
Attract.	−0.81	17	0.428	−0.37	−1.28	0.54	0.82	6	0.400	0.12	1.65	5	0.260	0.25	0.27	13	0.610	0.02	0.44
Accent.	0.39	17	0.704	0.18	−0.73	1.08	7.55	6	0.030	0.56	1.37	5	0.300	0.21	0.39	13	0.550	0.03	0.47
Worry	0.63	17	0.537	0.29	−0.62	1.19	3.95	6	0.090	0.40	0.06	5	0.810	0.01	1.34	13	0.270	0.09	0.68
Sex.Discomfort	1.00	17	0.332	0.46	−0.46	1.37	0.00	6	0.950	0.00	0.53	5	0.500	0.10	0.28	13	0.610	0.02	0.46
SCL−27-plus																			
Dep	1.21	17	0.242	0.56	−0.37	1.47	1.20	6	0.310	0.17	7.14	5	0.040	0.59	0.09	13	0.760	0.01	0.43
Veg	0.43	17	0.670	0.20	−0.71	1.10	1.31	6	0.300	0.18	0.15	5	0.720	0.03	5.35	13	0.040	0.29	3.61
Ago	0.76	17	0.461	0.35	−0.57	1.25	2.81	6	0.140	0.32	0.21	5	0.670	0.04	0.81	13	0.380	0.06	0.56
Sop	0.14	17	0.891	0.06	−0.84	0.96	0.31	6	0.600	0.05	3.56	5	0.120	0.42	0.70	13	0.420	0.05	0.56
Pain	0.90	17	0.382	0.41	−0.51	1.32	6.68	6	0.040	0.53	4.20	5	0.100	0.46	0.10	13	0.760	0.01	0.43
GSI	0.96	17	0.353	0.44	−0.48	1.35	0.50	6	0.510	0.08	3.36	5	0.130	0.40	1.34	13	0.270	0.09	0.85
LTDep	1.62	17	0.123	0.75	−0.20	1.67	10.40	5	0.020	0.68	0.02	4	0.890	0.01	1.88	11	0.200	0.15	1.14
f (suic. thoughts)	1.66	9	0.132	0.72	−0.22	1.64	0.48	6	0.510	0.07	0.34	6	0.580	0.05	2.20	13	0.160	0.14	1.12
f (suic. attempts)	1.00	8	0.347	0.47	−0.47	1.40	0.00	5	1.00	0.00	-	-	-	-	-	-	-	-	-
BDI																			
total score	0.67	17	0.512	0.31	−0.60	1.21	0.07	6	0.790	0.01	0.71	5	0.440	0.13	0.68	13	0.420	0.05	0.50

* Inferential statistics for univariate analysis. Admission differences were evaluated using independent samples *t*-test. In case of violated assumptions about homoscedasticity, *t*-tests with adjusted degrees of freedom (df) are reported. Changes from admission to end-of-treatment, and gender-based comparisons of change, were analyzed using univariate ANCOVAs with initial admission (baseline) levels and length of treatment as co-variates. See Methods for further details. Bold values indicate *p* < 0.05. ED = Eating Disorder, EOT = End-Of-Treatment, Adm. = Admission, AN = Anorexia Nervosa, BN = Bulimia Nervosa, BED = Binge Eating Dis-order, EDNOS = Eating Disorder Not Otherwise Specified, BMI = Body Mass Index, zBMI = age- and gender-standardized BMI-Scores, EDEQ = Eating Disorder Examination Questionnaire, eat. = eating, lax. = laxatives, exer. = exercising, FBeK = Fragebogen zur Beurteilung des eigenen Körpers, Attract. = Physical Attractiveness and Self-confidence, Accent. = Accentuation of Physical Appearance, SCL-27-plus = Symptom Checklist SCL-27-plus, Dep = depressive symptoms, Veg = vegetative symptoms, Ago = agoraphobic symptoms, Sop = sociophobic symptoms, GSI = global symptom severity index, LTDep = lifetime assessment for depressive symptoms, suic. = suicide, BDI = Beck Depression Inventory. *t*, *df*, *d*, *LL*, *UL*, *F*, *eta^2^*, *F*, *BFincl* are statistical elements.

**Table A3:** LME pairwise comparisons (women–men) for change in weight outcomes (zBMI).

ED Group *	DOT	*est.*	*SE*	*df*	*t*	*p*
AN	0	−0.07	0.04	422	−1.50	0.134
	7	−0.09	0.04	402	−2.02	0.044
	14	−0.11	0.04	393	−2.53	0.012
	21	−0.13	0.04	394	−3.02	0.003
	28	−0.16	0.04	405	−3.50	0.001
	35	−0.18	0.04	427	−3.94	0.000
	42	−0.20	0.05	460	−4.34	0.000
	49	−0.22	0.05	506	−4.70	0.000
	56	−0.24	0.05	565	−5.03	0.000
	63	−0.26	0.05	639	−5.31	0.000
BED	0	0.03	0.03	427	1.26	0.207
	7	0.06	0.03	403	2.08	0.038
	14	0.08	0.03	392	2.90	0.004
	21	0.10	0.03	395	3.69	0.000
	28	0.12	0.03	413	4.45	0.000
	35	0.14	0.03	445	5.14	0.000
	42	0.16	0.03	493	5.77	0.000
	49	0.19	0.03	559	6.32	0.000
	56	0.21	0.03	645	6.79	0.000
	63	0.23	0.03	755	7.20	0.000
BN	0	0.04	0.07	425	0.57	0.566
	7	0.06	0.07	400	0.81	0.416
	14	0.07	0.07	391	1.05	0.294
	21	0.09	0.07	396	1.28	0.201
	28	0.11	0.07	415	1.49	0.136
	35	0.12	0.07	450	1.69	0.092
	42	0.14	0.08	501	1.86	0.063
	49	0.16	0.08	572	2.01	0.045
	56	0.17	0.08	664	2.14	0.033
	63	0.19	0.08	780	2.24	0.025
EDNOS	0	0.14	0.10	419	1.41	0.159
	7	0.11	0.10	399	1.17	0.243
	14	0.09	0.10	390	0.92	0.360
	21	0.06	0.10	392	0.66	0.512
	28	0.04	0.10	404	0.40	0.693
	35	0.01	0.10	428	0.14	0.892
	42	−0.01	0.10	463	−0.11	0.909
	49	−0.04	0.10	511	−0.35	0.724
	56	−0.06	0.10	573	−0.58	0.564
	63	−0.09	0.11	652	−0.79	0.432

* DOT = day of treatment, LME = linear mixed-effects. Bold values indicate *p* < 0.05. *est.*, *SE*, *df*, *t* are statistical elements.
